# Microalgae as a safe food source for animals: nutritional characteristics of the acidophilic microalga *Coccomyxa onubensis*

**DOI:** 10.3402/fnr.v60.30472

**Published:** 2016-10-17

**Authors:** Francisco Navarro, Eduardo Forján, María Vázquez, Zaida Montero, Elisabeth Bermejo, Miguel Ángel Castaño, Alberto Toimil, Enrique Chagüaceda, Miguel Ángel García-Sevillano, Marisa Sánchez, María José Domínguez, Rosario Pásaro, Inés Garbayo, Carlos Vílchez, José María Vega

**Affiliations:** 1 Department of Environmental Biology and Public Health, Cell Biology, Faculty of Experimental Sciences, University of Huelva, Huelva, Spain; 2 Algal Biotechnology Group, CIDERTA and Faculty of Sciences, University of Huelva and Marine International Campus of Excellence (CEIMAR), Huelva, Spain; 3 University Hospital Complex of Huelva Juan Ramón Jiménez, Huelva, Spain; 4 CIDERTA Investigation Center, University of Huelva, Huelva, Spain; 5 Department of Chemistry and Materials Science, Faculty of Experimental Sciences, University of Huelva, Huelva, Spain; 6 Riotinto Hospital, Huelva, Spain; 7 Department of Physiology, Faculty of Biology, University of Seville, Seville, Spain; 8 Department of Plant Biochemistry and Molecular Biology, Faculty of Chemistry, University of Seville, Seville, Spain

**Keywords:** *Coccomyxa onubensis*, rats, safe food, nutraceuticals, hypolipidemic induction

## Abstract

**Background:**

Edible microalgae are marine or fresh water mesophilic species. Although the harvesting of microalgae offers an abundance of opportunities to the food and pharmaceutical industries, the possibility to use extremophilic microalgae as a food source for animals is not well-documented.

**Objective:**

We studied the effects of dietary supplementation of a powdered form of the acidophilic microalga *Coccomyxa onubensis* on growth and health parameters of laboratory rats.

**Method:**

Four randomly organized groups of rats (*n*=6) were fed a standard diet (Diet 1, control) or with a diet in which 0.4% (Diet 2), 1.25% (Diet 3), or 6.25% (Diet 4) (w/w) of the standard diet weight was substituted with dried microalgae powder, respectively. The four groups of animals were provided *ad libitum* access to feed for 45 days.

**Results:**

*C. onubensis* biomass is rich in protein (44.60% of dry weight) and dietary fiber (15.73%), and has a moderate carbohydrate content (24.8%) and a low lipid content (5.4%) in which polyunsaturated fatty acids represent 65% of the total fatty acid. Nucleic acids are present at 4.8%. No significant difference was found in growth rates or feed efficiency ratios of the four groups of rats. Histological studies of liver and kidney tissue revealed healthy organs in control and *C. onubensis*-fed animals, while plasma hematological and biochemical parameters were within healthy ranges for all animals. Furthermore, animals fed a microalgae-enriched diet exhibited a statistically significant decrease in both blood cholesterol and triglyceride levels. The blood triglyceride content and very low density lipoprotein-cholesterol levels decreased by about 50% in rats fed Diet 4.

**Conclusions:**

These data suggest that *C. onubensis* may be useful as a food supplement for laboratory animals and may also serve as a nutraceutical in functional foods. In addition, microalgae powder-supplemented diets exerted a significant hypocholesterolemic and hypotriglyceridemic effect in animals.

The health of people is linked directly to lifestyle, of which a balanced diet plays a fundamental role. Within the concept of what is meant by such a diet, nutraceuticals and functional foods have become progressively relevant. From the middle of the 20th century, large-scale microalgae cultivation has been carried out in regions with harsh climatic conditions where other crops cannot be grown, such as desert and coastal areas, and the obtained biomass used for biotechnological applications (1).

Microalgae such as *Chlorella* spp., *Dunaliella* spp., *Scenedesmus* spp., *Nannochloropsis* spp., *Tetraselmis* spp., *Spirulina* spp., and *Aphanizomenon flos-aquae* have been used as nutrient-dense foods and sources of nutraceuticals for functional foods (2–4). The use of microalgae for animal feed and aquaculture is of particular interest (5). While edible microalgae have been obtained from marine or freshwater mesophilic species, just a few attempts have been made to the present time to introduce the production of extremophilic microalgae, including *Dunaliella*, *Spirulina*, and *Galdieria* species (6, 7), to be used as a feed or food source, probably due to low growth rates and poor biomass productivity. However, recent studies have shown that acidophilic *Coccomyxa onubensis* can be phototrophically cultivated at pH 2.5 in minimum mineral medium, thereby reaching a moderate growth rate (8).

Issues associated with the use of microalgal biomass as a direct food source for animals include its high nucleic acid content and possible contamination, which raise concerns regarding potential toxicity and long-term effects on human health (3). Some authors indicate that the future use of microalgae biomass in the food industry will be as a source of nutraceuticals for functional foods rather than the direct use of such biomass. Microalgal biomass contains variable amounts of biological compounds that are beneficial to human and animal health, including lipids, polysaccharides, antioxidants, vitamins, minerals, and biomolecules with pharmaceutical activities (9–11). Some of these compounds are considered as functional ingredients in traditional foods, such as breakfast cereals, spreads, breads, cookies, brownies, energy bars, mayonnaises, gelled dessert, pastas, emulsions, ice creams, and beverages (12).

Several polyhydroxysterols from microalgae have also been found to display cytotoxic and anticancer activities (13). Polysaccharides belong to a large family of highly diverse chemical compounds, some of which have been described to stimulate the human immune system and/or have potential biomedical applications (14). Therefore, there is no doubt concerning the high potential value of microalgal biomass as a possible source of nutraceuticals for functional foods.

Because the natural habitat of *C. onubensis* supports high irradiation and oxidative conditions, physicochemical and nutritional parameters can be adequately fixed to produce biomass with a high carotenoid (mainly lutein) and polyunsaturated fatty acid (PUFA) content (15). As such, *C. onubensis* might be a good model to investigate the potential role of an acidophilic photosynthetic microalga to be used in animal feed. This study was undertaken to elucidate the effects of microalgae-supplemented diets on the health of laboratory rats. Hematological and biochemical parameters were analyzed in parallel with histological studies, with results demonstrating no adverse effects on rat health. In addition, *C. onubensis–*supplemented diets exhibited a potent hypocholesterolemic and hypotriglyceridemic effect in experimental animals.

## Materials and methods

### Microalgal biomass production

The microalga *C. onubensis* (SAG 2510) (16), deposited at the Culture Collection of Algae at Goettingen University, was bulk produced in 400 L transparent plastic bags of 60 cm diameter and 2.1 m height. The bags were maintained indoors at 20°C, filled with K9 culture medium (adjusted to pH 2.5), and bubbled with CO_2_-enriched air (5% v/v) *via* air diffusers placed at the bottom of the bags. The cultures were illuminated with white fluorescent light at 200-µmol photons/m^2^s at the bag surface. The cells were harvested by continuous flow centrifugation at 8,400 rpm using an industrial centrifuge (GEA Westfalia Separator model KA-6, Oelde, Germany), and the collected pellet was washed twice with deionized water. The biomass was dried in an oven with fan-assisted circulation (JP Selecta DRY-BIG 2002972, Barcelona, Spain) and converted into a powder of grain size <100 µm using a vibratory disc mill (Retsch GmbH RS100, Haan, Germany). The powder was then vacuum packed and stored at −80°C until use.

### Chemical analysis of the *C. onubensis* biomass

The main components of the algal biomass were analyzed by the Central Services Unit of the University Pablo de Olavide (Seville, Spain). To determine the fatty acid composition of the biomass, the acid catalyzed transesterification of extracted glycerides was carried out in flasks according to the following procedure: the standard reaction mixture containing glycerides, methanol, and concentrated sulfuric acid (5% v/v) was heated at 70°C for 3 h, then cooled and treated with hexane and water. The mixture was separated by centrifugation (2,000 rpm, 10 min) into two layers, the upper lipidic layer of which was washed with water until the washing water was pH neutral. Analysis of the resulting fatty acid monoester (FAME) composition of this hexane layer was carried out using an Agilent 7890A gas chromatography unit (Agilent Technologies, Wilmington, DE, USA) equipped with flame ionization detector. Samples (1 µL) were injected into an OMEGAWax-fused silica capillary column (30 m, 0.32 mm id, and 0.25 µm film thickness) in which the flow rate of the carrier gas helium was constant at 1.5 mL/min and a split ratio of 20:1 was used. A solvent delay period of 1.5 min was assigned. The injector temperature was 100°C, and the detector temperature was maintained at 200°C. The oven temperature was raised from 80 to 140°C at 5°C/min, then increased up to 170°C at 4°C/min, and maintained for 2 min at that temperature. It was then raised to 190°C at 1°C/min and maintained for 2 min, and finally, the oven temperature program was increased to 200°C. Individual FAMEs were identified by comparing their retention times with those of a mixed FAME standard (FAMEs MIX C4-C24 SUPELCO Analytical, Bellefonte, PA, USA). Concentrations of FAMEs in the injected hexane solution were quantified by comparing their peak areas with those obtained from the standards of known concentration. Fatty acid composition was calculated as the percentage of total fatty acids in the volume of hexane.

### Experimental diet preparation

The experimental diets used in this work were based on conventional rodent chow pellets from Harlan Laboratories, Inc. (Indianapolis, IN, USA), which were grounded into powder using a jaw crusher (Retsch GmbH BB200) and a vibratory disc mill until the grain size was <100 µm. The four diets were prepared as follows: Diet 1, control: composed of standard diet powder only; Diet 2: 4 mg of microalgae powder were mixed with 996 mg of standard diet powder; Diet 3: composed of 12.5 mg microalgae powder plus 987.5 mg standard diet powder; and Diet 4: 62.5 mg microalgae powder plus 937 mg standard diet powder. In all cases, the powders were mixed homogeneously, reconstituted with distilled water in a kneader (Fimar AM1, Rimini, Italy), and then made into pellets again with an extruder (Fimar MPF4). The four reconstituted pellet diets were dried in an oven with fan-assisted circulation to obtain the same degree of humidity as the original standard rodent diet. Dried pellets were stored at 4°C under vacuum until used.

According to equivalences shown in Table 1, the amount of microalga used in these experimental diets was comparable to a human consumption of 20.4–255 g/day microalgae powder, which would be sufficient quantities to enable information about toxicity to be obtained.

**Table 1:** Relationship of microalga powder in rat diets and its corresponding equivalence in human diets

Diet	Alga amount in rat diet (%)	Alga consumption by rats (mg/day)	Equivalent alga consumption by 68 kg person (g/day)
1	0	0	0
2	0.4	60	20.4
3	1.25	187.5	63.8
4	6	750	255

Calculations were made for an average size (200 g) rat consuming 15 g/day of feed (26).

### Animal handling

Experiments were performed on 4-week-old Long Evans male rats (*n*=24) weighing 130–140 g, obtained from the Charles River Laboratories (St. Germain-Nuelles, France). Animals were handled in accordance with Directive 8609/CEE of the European Community Council and Spanish Legislation (R.D. 53/2013). The protocols used in this work were approved by the Ethics Committee of the University of Huelva (Spain). Animals were allowed to acclimatize for 5 days with free access to food (Diet 1) and water under controlled conditions of temperature (22.0±1.3°C) and a 12 h light–dark cycle prior to the start of experiments. They were then randomly distributed into four groups of six rats each, with similar mean weights, housed individually, and fed with one of the described diets (Diet 1, Diet 2, Diet 3, and Diet 4). They were weighed every second day, and biochemical and histological analyses were performed on all animals after a period of 45 days of *ad libitum* feeding.

### Hematological and biochemical analyses

Rats were fasted for 8 h and anesthetized with inhaled isoflurane prior to sacrifice. Following a cardiac puncture, blood was collected from the left ventricle in a 2 mL glass BD Vacutainer K3 EDTA tube (Becton, Dickinson and Company, Franklin Lakes, NJ, USA) and processed immediately according to the manufacturer's instructions for hematological studies. The hematological analysis was carried out on a Sysmex XT-4000i automated hematology analyzer (Sysmex America, Inc., Lincolnshire, IL, USA). To study the erythrocyte morphology, blood smears were prepared following the procedure of Bain et al. (17), stained with Hemacolor^®^ staining kit (Merck Millipore, Darmstadt, Germany), and examined under a Nikon Eclipse E400 microscope (Nikon, Tokyo, Japan).

Samples for serum biochemistry determinations were collected in 2 mL tubes with the Advanced BD Vacutainer SST II gel separator and suction system. Blood samples were first cooled in a refrigerator and protected from light for 60 min to allow clot retraction, and serum obtained after centrifugation at 4,000 rpm for 30 min at 4°C. Parameter determinations were carried out on a Sysmex XT-4000i automated hematology analyzer (Sysmex America, Inc.). Enzyme activities were determined on a Cobas 8000 modular analyzer (Roche Diagnostics, Basel, Switzerland) according to the manufacturer's instructions. Plasma total cholesterol (TC) and triglyceride concentrations were determined enzymatically on the Cobas 8000 modular analyzer according to the manufacturer's instructions. The same instrument was also used to analyze high density lipoprotein (HDL)- and very low density lipoprotein (VLDL)-cholesterol contents following the manufacturer's instructions in each case.

### Histological analysis

Liver and kidney tissues from animals in the different group were extracted, weighed, cleaned with 0.9% (w/v) NaCl solution, and fixed in 4% neutral buffered formalin. This was followed by dehydration in ascending grades of alcohol for 51 h, xylene for 1 h, and finally embedding in paraffin wax. Liver and kidney sections (4 mm thickness) were obtained with a Leica Leitz 1512 precision rotary microtome (Leitz, Wetzlar, Germany) and stained with hematoxylin and eosin for histological examination. Slide-mounted sections were observed under light microscope (Nikon Eclipse E400 microscope) for the evaluation of tissue integrity.

### Statistical analyses

Statistical analyses were conducted by using the SPSS version 19 statistical analysis package. The data of the four dietary groups were analyzed by a non-parametric test (Kruskal–Wallis). Differences were accepted as significant for values of *p*<0.05.

## Results

### Effect of *C. onubensis* biomass on rat body weights

The composition of *C. onubensis* biomass is indicated in Table 2. Of particular interest from a nutritional point of view was the high protein (44.60% of dry weight) and dietary fiber (15.73%) content of the material, together with a moderate carbohydrate content (24.80%), and a low lipid (5.40%) and nucleic acids (4.8%) content. An additional dietary advantage of this biomass is its low monosaccharide and disaccharide content (0.1%), and high PUFA content (65% of total fatty acid content).

**Table 2:** Chemical composition of dry biomass from *C. onubensis*

Parameter	Fraction composition (%)	Sub-fraction composition (%)
Proteins	44.60	–
Carbohydrates	24.80	–
Soluble sugars	–	4.11
Dietary fiber	15.73	–
Triglycerides	5.40	–
Saturated FA	–	17.55
Monounsaturated FA	–	17.45
Polyunsaturated FA	–	65.00
Nucleic acids	4.8	–

The dry weight represents 24.35% of the biomass. Numbers are per cent of dry weight. Parameters were determined as indicated in Materials and Methods. FA, fatty acids.

The four groups of rats exhibited constant weekly weight gain, which at the end of the experiment ranged from 210±8.08 g for control animals (Diet 1) to 205±8.46 g for the Diet 4 group. Rats did not show any visible physiological or behavioral alterations over the experimental period. The ratio of food consumed (g)/weight gained (g) (feeding efficiency) was between 5.04±0.20 (Diet 1, control) and 4.40±0.11 (Diet 4) (Table 3), which is within the normal range for rats.

**Table 3:** Anthropometric data for control rats and rats fed powdered *C. onubensis* in the diet

Anthropometric data	Diet 1	Diet 2	Diet 3	Diet 4
Initial weight (g)	134±4.64	137±2.38	125±4.64	123±3.35
Final weight (g)	344±6.62	338±7.72	336±8.76	328±7.77
Weight gain (g)	210±8.08	201±8.07	211±9.91	205±8.46
Feed consumption (g)	1,058±43.38	1,016±40.65	939.5±43.22	902±37.88
Feed efficiency ratio	5.04±0.20	5.06±0.22	4.45±0.17	4.40±0.11
Liver weight (g)	10.48±0.55	9.68±0.36	9.78±0.24	9.85±0.64
Relative weight (g/100 g bw)	3.00	2.86	2.90	3.00
Kidney weight (g)	2.13±0.28	2.04±0.35	1.97±0.42	2.01±0.38
Relative weight (g/100 g bw)	0.60	0.60	0.50	0.60

Each value is expressed as the mean±SE (*n =*6) in all groups. Results were statistically analyzed with Kruskal–Wallis test. No significant differences were observed at the *p<*0.05 level among the different diets. bw, body weight.

### Histological studies of liver and kidney

At the end of the experiment, each rat was sacrificed, and its liver and kidneys extracted and studied. Freshly excised organs showed a normal aspect in all groups and weighed 10.48±0.55 g for liver and 2.13±0.28 g for kidneys (Table 3). The relative weights of the liver and kidney tissues (g/100 g body weight) were similar for all groups of animals; at around 3.00 for liver and 0.60 for kidney in the control and Diet 4 groups (Table 3).

**Fig. 1:**
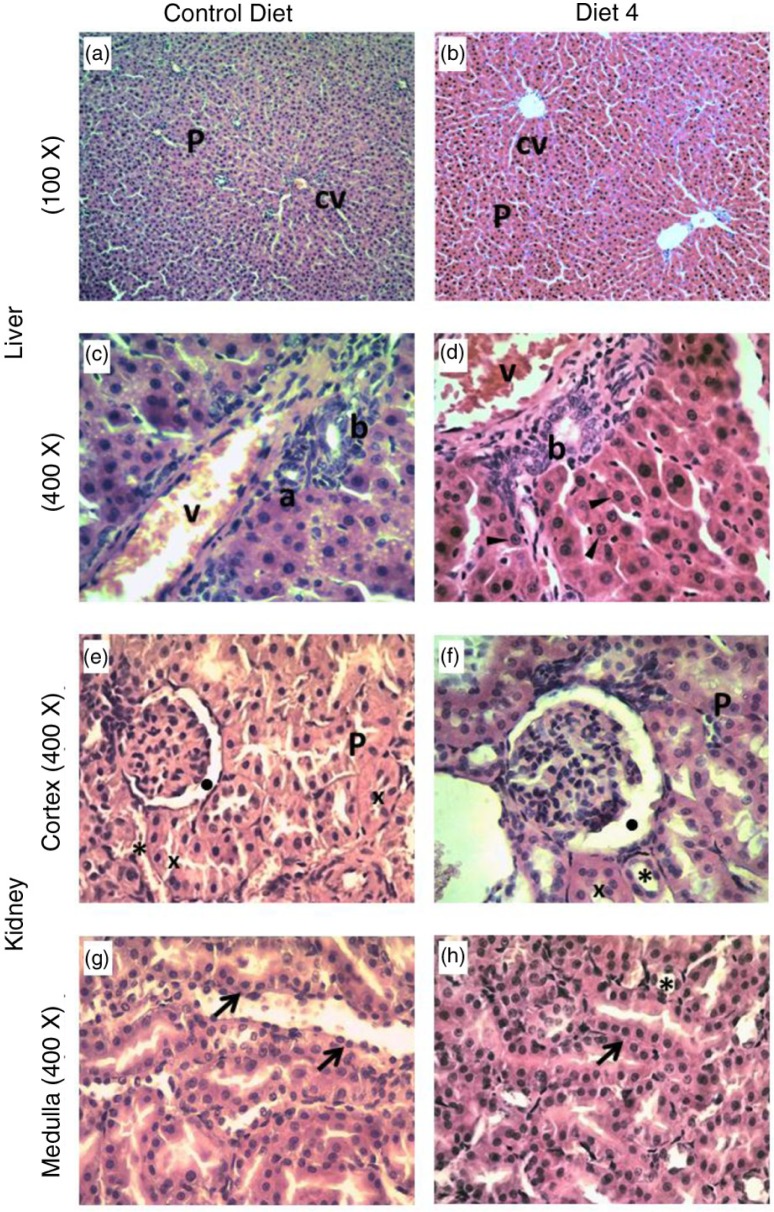
Hematoxylin and eosin stained sections of rat liver and kidney from experimental rats. Liver: a,b. Hepatocyte architecture in parenchyma (P) and central vein (cv). c,d: Higher magnification of hepatic sections where the portal triad it can be observed: normal portal vein (v), artery (a), and bile duct (b) without occlusions. d. Black arrow heads indicate hepatocyte nuclei. Kidney: e-h. Sections of the cortex (black dot) and convoluted proximal (x) and distal (asterisks) tubules are shown in e,f. g,h: Medulla. Longitudinal section of tubule epithelium (black arrows).

The thickness and configuration of the trabeculae, the presence of regenerative changes, and an initial assessment of fibrosis in the liver were carried out, with no pathogenic changes observed for any groups (Fig. 1a–d). In addition, parameters indicative of degeneration and substance deposition, such as ballooning degeneration (swelling), presence of cholestasis, and of macro and micro vesicular steatosis, were found to be normal in all groups. All of the sections studied showed a healthy hepatocyte architecture in the parenchyma and central vein, with similar degrees of steatosis in the different groups of rats. However, striking alterations were identified in the chromatin pattern of hepatocyte nuclei from the Diet 4 group (Fig. 1d), which did not affect the health of animals. Likewise, the cytoplasm presented an increased uptake of hematoxylin in some areas without signs of cell swelling. No hepatocellular damage such as tissue necrosis or apoptosis was found. Predominant cell types and inflammation parameter levels were also found to be normal (Fig. 1a and b) (18).

With respect to kidney tissue, attention was focused on the glomerulus, where we looked for the possible presence of sclerosis, inflammatory infiltrates, thrombi, and/or deposits. We also examined the interstitium and tubules, where we looked for the presence of atrophy, inflammatory infiltrates, necrosis, and deposits. All of studied sections in the different groups of animals presented a normal histology, with no signs of degeneration (Fig. 1e–h) (18). In cortical regions of the kidney, where the Bowman's capsule is located, the capsular space and podocytes were distributed in a normal fashion, independently of the diet received by the rat (Fig. 1e and f). The proximal convoluted and distal tubules also showed a normal epithelial cell distribution. Most of the tubules in the medulla (Fig. 1g and h) presented a longitudinal section and an intact collecting tubule epithelium. Attention was paid to the structure of blood vessels to identify the possible presence of vasculitis, thrombi, hyalinization, or sclerosis, but all of the studied histological sections appeared normal (18), thereby indicating a lack of toxicity of the microalgae powder-containing diets.

### Blood and biochemical parameters

Table 4 shows hematological parameters for the four experimental animal groups. No significant differences were identified, and the blood profiles were typical of healthy animals. In relation to biochemical parameters, the plasma total protein content was significantly lower for the Diet 3 and Diet 4 groups, whose diet had a higher percentage enrichment of algae. The plasma glucose concentration, 115.50 mg/dL, in rats fed with the Diet 4 was higher than the control group (70.18 mg/dL, Diet 1) (Table 5). However, values did not exceed 135 mg/dL, which is considered the upper limit for healthy rats. Levels of other plasma parameters such as albumin, creatinine, ferritin, bilirubin, and the Ca^2+^, Na^+^, K^+^, Cl^−^, and Fe^2+^ ions were similar across the different groups of rats, and within normal ranges (Table 5).

**Table 4:** Hematological parameters of rats fed control or *C. onubensis–*supplemented diets

Parameter	Diet 1	Diet 2	Diet 3	Diet 4
Hemoglobin (g/dL)	14.58±0.99	14.78±1.1	15.88±0.11	16.08±0.05
Hematocrit (%)	44.43±2.93	44.25±3.12	47.38±0.46	48.00±0.36
Erythrocytes (cells×10^−6^/µL)	8.66±0.60	8.24±0.57	8.76±0.13	8.61±0.03
Leucocytes (cells×10^−3^/µL)	5.57±0.89	5.35±1.04	8.23±1.89	6.65±1.02
Lymphocytes (%)	84.75±1.89	90.00±2.64	85.75±2.06	89.75±0.63
Platelets (cells×10^−3^/µL)	584.67±18.46	618.33±29.80	665.00±10.13	779.00±14.12

Data obtained at the end of the experiment (45 days). Each value is expressed as the mean±SD (*n=*6 per group). Results were statistically analyzed with Kruskal–Wallis test. No significant differences were observed at the *p<*0.05 level among the different diets. More details in Materials and Methods section.

**Table 5:** Biochemical parameters of rats fed control or *C. onubensis–*supplemented diets

Parameter	Diet 1	Diet 2	Diet 3	Diet 4
Total protein (g/dL)	6.05±0.05	6.18±0.02	5.45±0.31*#	5.65±0.35*#
Albumin (g/dL)	4.40±0.11	4.48±0.11	4.23±0.25	4.28±0.23
Ca^2+^ (mg/dL)	10.40±0.50	10.40±0.5	10.19±0.19	10.40±0.32
Na^2+^ (mEq/L)	145.00±0.82	146.00±1.78	144.25±1.65	147.00±0.91
K^+^ (mEq/L)	4.43±0.19	4.43±0.19	5.26±0.69	4.22±0.38
Cl^−^ (mEq/L)	90.13±1.07	98.13±1.07	99.15±0.65	99.88±1.62
Glucose (mg/dL)	70.18±4.97	99.68±7.63	115.50±14.47	103.03±15.44
Creatinine (mg/dL)	0.32±0.02	0.32±0.02	0.32±0.02	0.27±0.03
Fe ^2+^ (µg/dL)	128.90±6.44	136.33±7.16	137.45±7.89	139.58±11.89
Ferritin (ng/mL)	185.30±5.52	177.93±2.81	174.38 ±9.97	152.70±25.70
Bilirubin (mg/dL)	0.04±0.01	0.07±0.01	0.10±0.02	0.08±0.03

Data obtained at the end of the experiment (45 days). Each value is expressed as the mean±SD (*n=*6 per group). Results were statistically analyzed with Kruskal–Wallis test. The only significant difference observed was for total protein.

* *p*<0.05 compared with the control group.

# *p*<0.05 compared with other groups. No significant difference was observed at the *p*<0.05 for other parameters. More details in Materials and Methods section.

**Fig. 2:**
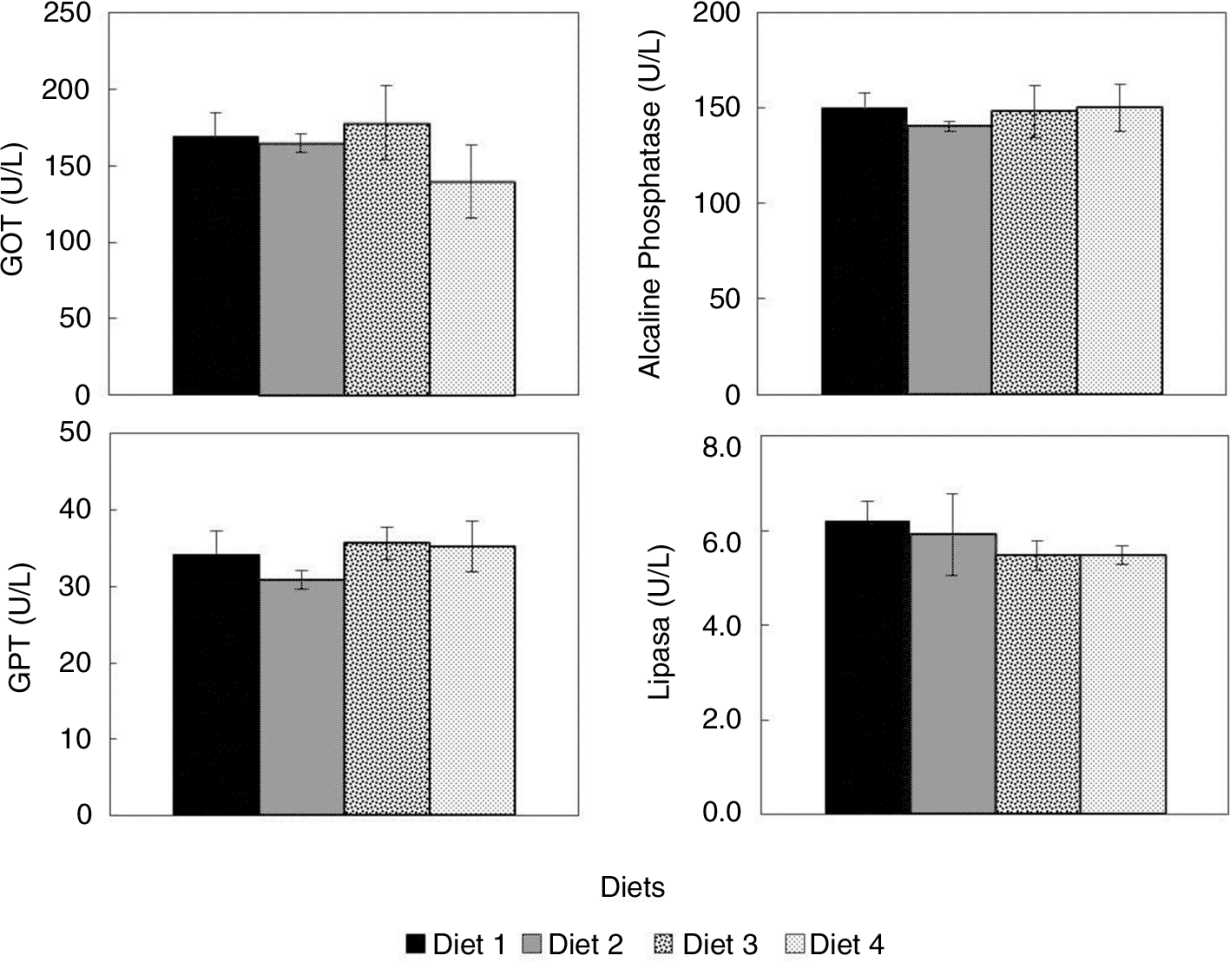
Enzyme activities in plasma from rats fed diets supplemented with *C. onubensis* biomass. Experimental conditions are described in the Materials and Methods. GOT, glutamate-oxaloacetate transaminase; GPT, glutamate-pyruvate transaminase. Each value is expressed as the mean±SD (*n=*6 per group). Results were statistically analyzed with Kruskal–Wallis test. None of the diets (Diet 2, Diet 3, and Diet 4) showed significant differences at the *p*<0.05 level with respect to each other, or relative to the control (Diet 1).

Diets supplemented with *C. onubensis* powder did not influence blood transaminase or alkaline phosphatase activity levels in rats. Glutamate-oxaloacetate transaminase (GOT) and glutamate-pyruvate transaminase activities in plasma were unchanged in rats fed supplemented diets compared with controls (Fig. 2). Finally, only a small decrease was observed in the plasma lipase activity level from rats fed the algae-supplemented feed with respect to control (Fig. 2).

**Fig. 3:**
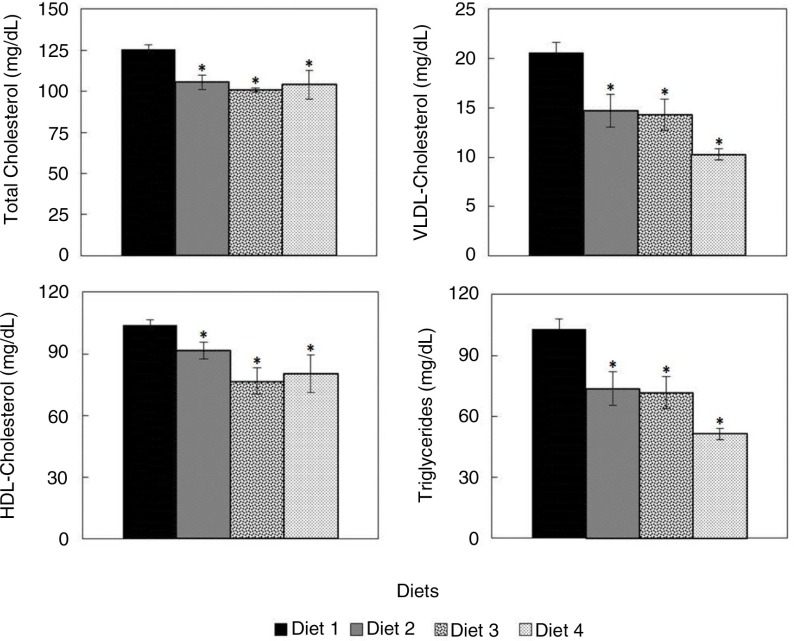
Lipid profiles of rats fed with diets supplemented with *C. onubensis* biomass. Experimental conditions are described in the Materials and Methods section. Each value is expressed as the mean±SD (*n=*6 per group). Results were statistically analyzed with Kruskal–Wallis test. *p<0.05 compared with the control group. None of the diets (Diet 2, Diet 3, and Diet 4) showed significant differences at the *p*<0.05 level with respect to each other. HDL=High density lipoprotein. VLDL=Very low density lipoprotein.

Importantly, *C. onubensis* powder-supplemented diets induced a significant decrease in plasma triglyceride contents, which reached 50% in Diet 4-fed rats compared to control rats (Diet 1). A similar decrease was observed in the plasma VLDL-cholesterol content, while TC content decreased by around 20% (Fig. 3). On contrary, HDL-cholesterol content decreased significantly by 25%, while the low density lipoprotein (LDL) cholesterol content increased by 30% (not shown).

## Discussion

The composition of *C. onubensis* biomass (Table 2) suggests that it has nutritional properties that may be more beneficial than other microalgae used in animal foods, where protein contents range from 1 to 65%, carbohydrate from 15 to 50%, and fat from 3 to 48% (4, 19). Biomass from *Chlorella vulgaris*, one of the most used species of microalgae, employed as a food supplement contains 28.0% protein, 49.5% carbohydrate, 17.5% lipid and 4.5% nucleic acids (20). Microalgal lipids are particularly interesting because they contain high levels of PUFAs such as docosahexaenoic (DHA) and eicosapentaenoic (EPA) acids, as well as carotenoids, such as astaxanthin and lutein, and other antioxidants (21). *C. onubensis* biomass is particularly rich in lutein and β-carotene, which have high antioxidant activities and are considered very useful as a source of nutraceuticals (8). Antioxidant phenolic compounds and vitamins (A precursor, E, and B group) are also produced by microalgae and accumulate at different levels depending on the cultivation conditions (4, 9). Further studies are necessary to examine ways to increase the antioxidant phenolic compounds and vitamin contents in *C. onubensis* biomass, and thereby to improve the properties of this material. The content in nucleic acids of *C. onubensis* is 4.8%, which is within the average nucleic acid content for most of microalgae, including those which are used as food for humans (22). This value should be compared with 1.5 and 2.2% of nucleic acids content found in beef and beef liver, respectively. It is generally considered that the long-term maximum acceptable daily intake of nucleic acid is about 4.0 g/day for an adult (23).

In general, the use of powdered microalgae in diets here was well tolerated by rats, as indicated by the similar weight gains in the four experimental groups over the course of the experiment (Table 3). In this context, a decrease in the body weight of animals has been observed with other microalgae-supplemented diets such as *Isochrysis galbana* and *Nannocholopsis oculata* (24), and the red microalga *Porphyridium* sp. (25). In contrast, rats fed a powdered form of the cyanobacteria *A. flos-aquae* showed a body weight increase of around 10% with respect to the control group (26).

Histological studies, along with hematological and biochemical analyses demonstrated that the *C. onubensis* powder-supplemented diets were not associated with any toxic side-effects to animals. The observed plasma levels of albumin and creatinine suggested that the four groups of animals had a good nutritional status and good renal function, while plasma transaminase activity levels demonstrated that the liver status of animals was good. In line with these observations, Ekmay et al. (27) observed a lack of toxicity in laying hens fed diets supplemented with biomass from the green microalga *Desmodesmus* spp. or the diatom *Staurospira* spp. The high phenolic compound content in *Spirulina platensis* biomass seems to exert a hepatoprotective effect in rats (28).

Previous findings concerning the plasma glucose concentration in animals fed on microalgal biomass are contentious. For example, *I. galbana* induced a decrease in plasma glucose and cholesterol values, whereas consumption of the microalga *N. oculata* showed no benefit for normal and diabetic rats (24). The moderate hyperglycemic effect induced by *C. onubensis* powder in rats in this study (Table 5) had no apparent consequences on animal health status. This effect cannot be directly attributed to the microalgae powder because soluble carbohydrates found in this biomass were very low (Table 2).

The *C. onubensis* biomass induced a potent hypolipidemic activity in rats, which suggests that this material may have been used as a source of nutraceuticals in functional foods and/or have pharmacologic applications. It is important to note that, in humans and rodents, the liver is the primary site for *de novo* lipogenesis, where glucose is the main carbon precursor for this process. However, a principle difference between these species is that carry out the majority of cholesterol in the plasma of rodents is bound to HDL-lipoproteins, whereas in humans it is bound to LDL-lipoproteins (29). In animals, ProAlgaZyme, an infusion of fermented algae biomass, induces a significant reduction in body weight, body fat, TC, LDL-cholesterol, and triglycerides and is accompanied by a significant increase in HDL-cholesterol levels. This infusion is well tolerated by animals and does not cause significant side effects (30). Results obtained with *C. pyrenoidosa* are particularly interesting, as this microalga was shown to prevent hyperlipidemia and atherosclerosis in rats and hamsters fed chronically with a high-fat diet (31). A study performed on chickens demonstrated that both 5 and 10% levels of *Porphyridium* sp. biomass supplemented in the diet were effective in lowering cholesterol levels, while a trend toward lower cholesterol in eggs was also observed (32). The next step in our research will be to examine the hypolipidemic effects of *C. onubensis* on rats fed a cholesterol-rich diet.

Identification of the active constituents that give rise to the hypolipidemic activity of *C. onubensis* must be further investigated. In this context, PUFAs have been reported to reduce blood LDL-cholesterol and HDL-cholesterol levels (33). Van Beelen et al. (34) suggested that omega-3 fatty acids lowered blood LDL-cholesterol levels, producing similar protective effects to those of fish oil against heart disease, atherosclerosis, cancer, and diabetes. The fatty acid content of *C. onubensis* is rich in PUFAs (see Table 2), which could be responsible for the observed lipoprotein profile in rats, although it is possible that the presence of other components could also be involved. Chen et al. (35) concluded that microalgal lipids are the active compounds responsible for the triglyceride- and cholesterol-lowering activity induced in hamsters. Algal-DHA acid induced a significant dose-related decrease of triglycerides and cholesterol in rats fed a high fructose diet (36). In this context, it is important to note that algae rich in n-3 fatty acids have been used in feed supplements for dairy cattle (37).

Our study shows that the acidophilic microalga *C. onubensis* provides a good source of dietary fiber, which may help to promote hypocholesterolemic and hypotriglyceridemic outcomes in rats due to its high PUFA content. Polysaccharides and dietary fiber have also been demonstrated to be involved in the hypolipidemic effect of *Porphyridium* (25). Animal studies have shown that the consumption of dietary fiber is associated with changes in lipid metabolism (9). In addition, soluble polysaccharides and biomass obtained from the red microalga *Porphyridium* sp. altered the intestinal morphology and reduced serum cholesterol in rats (25). Several health benefits derived from the presence of algal polysaccharides in the diet have also been reported in humans (38, 39). On contrary, sterol extracts from the alga *Schizochytrium* sp. were as effective as β-sitosterol in reducing plasma cholesterol concentrations (40).

## Conclusions

Based on histological, hematological, and biochemical analyses, this study shows that a diet enriched with *C. onubensis* biomass is non-toxic to rats. No negative effects were observed on body weight, physiological or morphological parameters over the course of the experimental period. *C. onubensis* biomass also had significant hypocholesterolemic and hypotriglyceridemic effects in rats. As such, *C. onubensis* biomass may serve directly as a functional food for animals or as source of nutraceutical and pharmacological compounds.
